# Supplementary material for: Spermidine-mediated hypusination of translation factor EIF5A improves mitochondrial fatty acid oxidation and prevents non-alcoholic steatohepatitis progression
Source: Nat Commun. 2022 Sep 3;13:5202. doi: 10.1038/s41467-022-32788-x (PMC9440896; doi:10.1038/s41467-022-32788-x)

Supplementary Figure 1

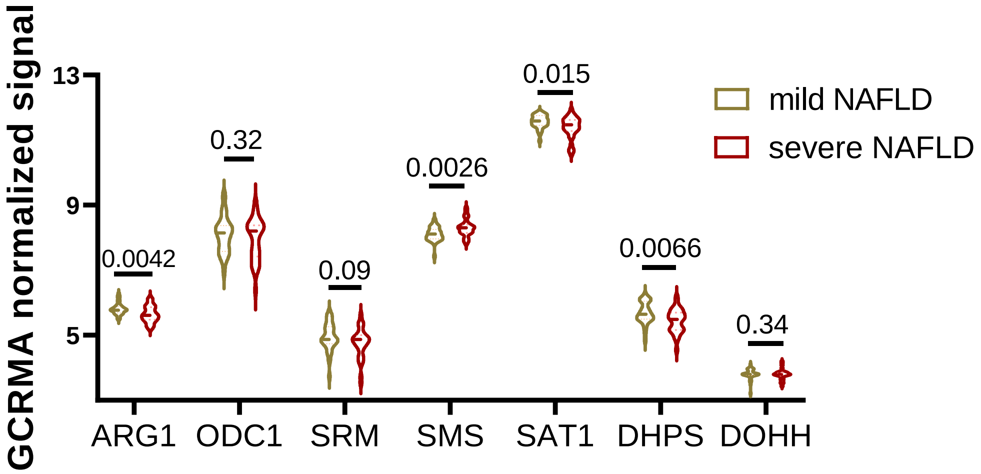

Fig. S1. Violin plots showing mRNA levels of genes involved in endogenous polyamine biosynthesis and EIF5A hypusination in patients with mild NAFLD (fibrosis stage 0-1; n=35) and severe NAFLD (fibrosis stage 3-4, n=31). GCRMA normalized signal was retrieved from microarray dataset (GSE49541, https://www.ncbi.nlm.nih.gov/geo/query/acc.cgi?acc=GSE49541)[47]. Significance was calculated by two-tailed Student's t-test or Mann-Whitney U-test, as appropriate. Source data are provided as a Source Data file.

Supplementary Figure 2

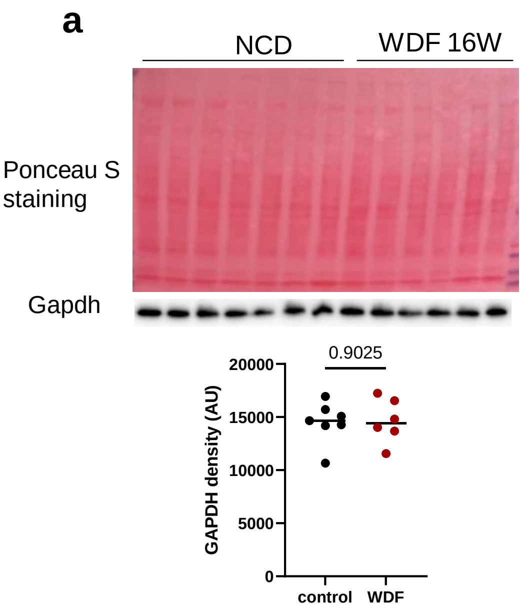

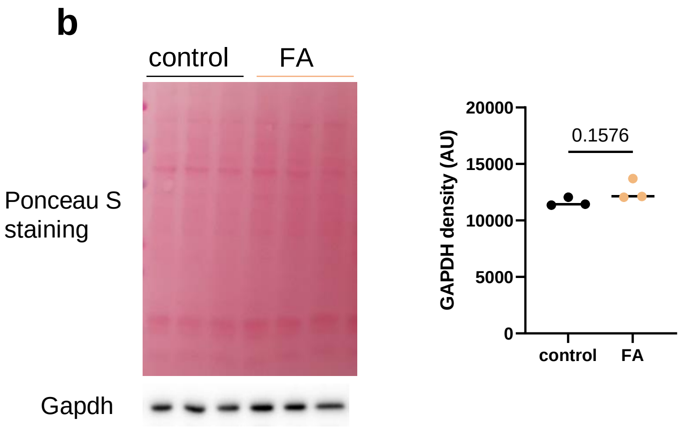

Fig. S2. Ponceau S staining of PVDF membrane after transfer, Western blot and density of GAPDH. (a) Liver protein lysate from mice fed on NCD (n=7), WDF (n=6). (b) AML12 cell lysate treated with or without FA (palmitic acid 0.6 mM, oleic acid 0.17 mM) for 48 h. (n=3). Source data are provided as a Source Data file.

Supplementary Figure 3

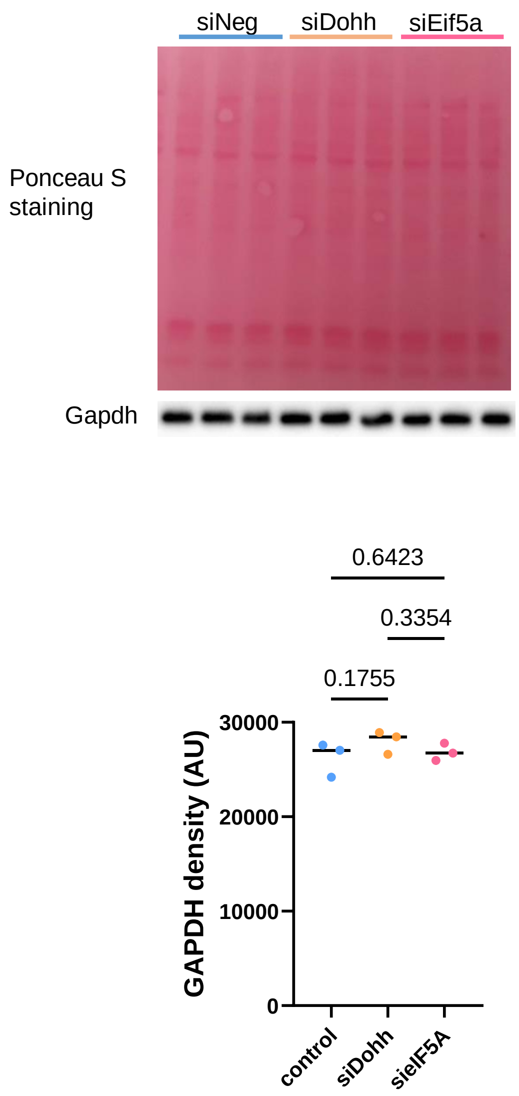

Fig. S3. Ponceau S staining of PVDF membrane after transfer, Western blot and density of GAPDH. AML12 cells were transfected with 20 nM of negative (siNeg), Dohh (siDohh) or Eif5a (siEif5a) siRNA for 48 h. (n=3). Source data are provided as a Source Data file.

Supplementary Figure 4

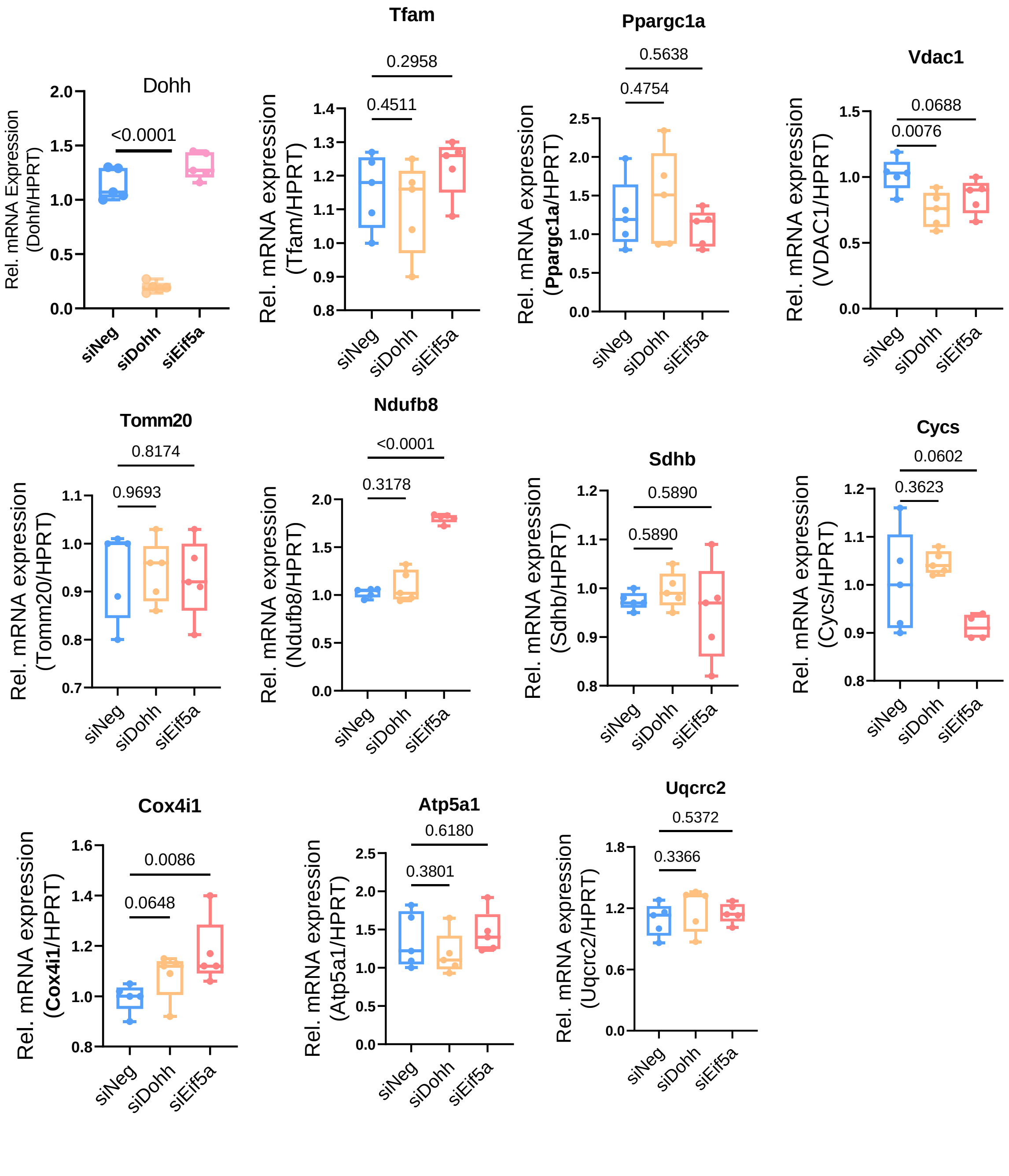

Fig. S4. Knockdown of Dohh or Eif5a didn't affect the mRNA expression of a panel of mitochondrial proteins. mRNA expression of makers for mitochondrial biogenesis and mitochondrial proteins in Dohh or Eif5a knockdown AML12 cells. Cells were transfected with 20 nM of negative (siNeg), Dohh (siDohh), or Eif5a (siEif5a) siRNA for 48 h. (n=5). Data are shown as box-and-whisker with median (middle line), 25th–75th percentiles (box), and min–max values (whiskers). Significance was calculated by One- Way ANOVA or Kruskal-Wallis test, as appropriate. Source data are provided as a Source Data file.

Supplementary Figure 5

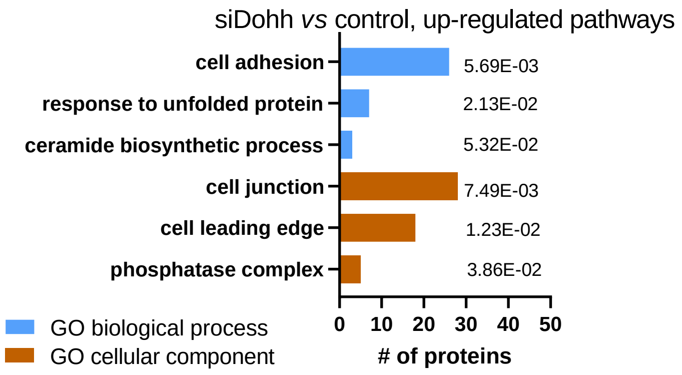

Fig. S5. Proteomics and gene ontology (GO) enrichment analysis (with corrected p-value indicated on the bar) of up-regulated proteins in siDohh *vs* control cells. A Bonferroni correction was applied to correct for multiple testing. Source data are provided as a Supplementary Data file.

Supplementary Figure 6

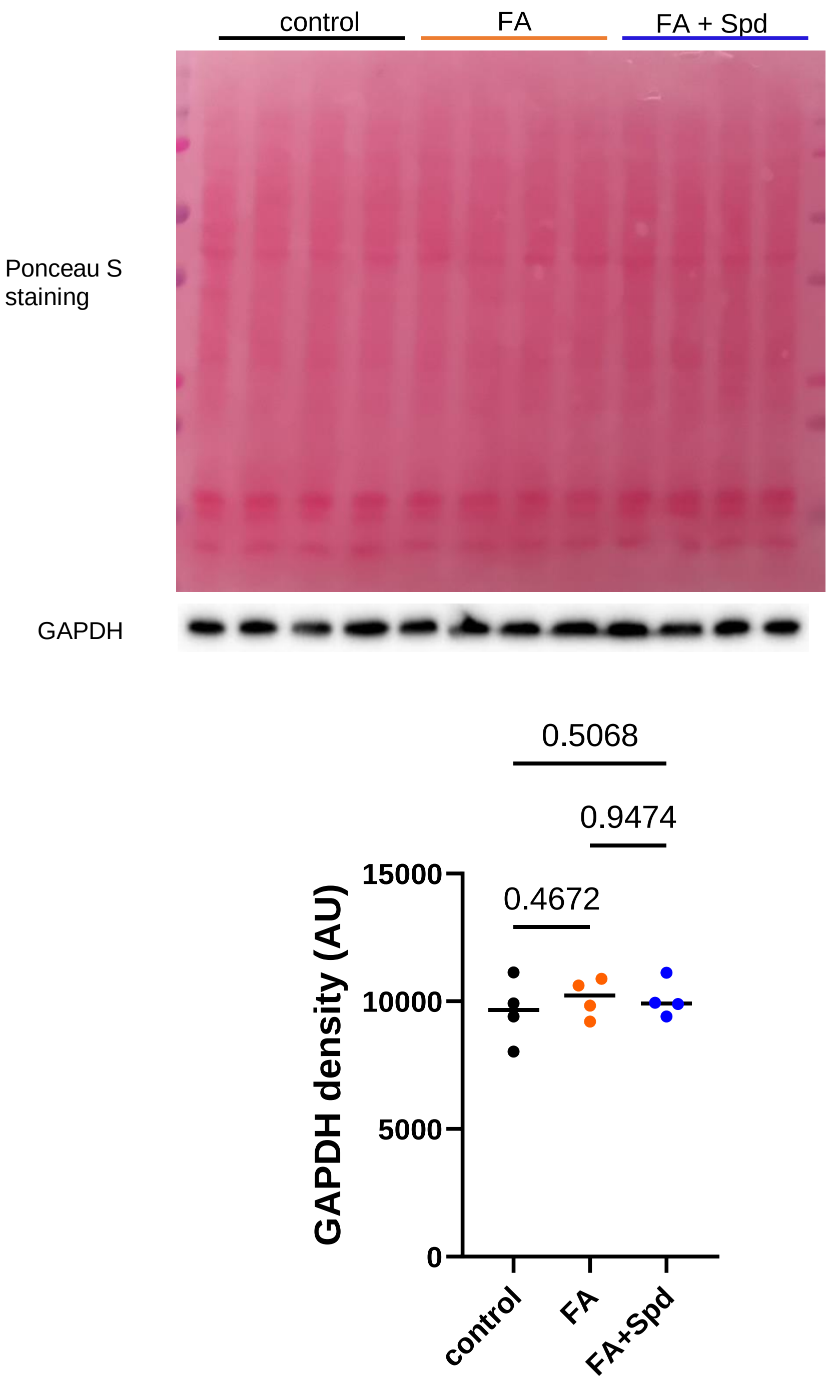

Fig. S6. Ponceau S staining of PVDF membrane after transfer, Western blot and density of GAPDH. AML12 cell lysate was harvested 48 h after treatment with or without FA (palmitic acid 0.6 mM, oleic acid 0.17 mM), FA + Spd (100 µM) for 48 h.(n=4). Source data are provided as a Source Data file.

Supplementary Figure 7

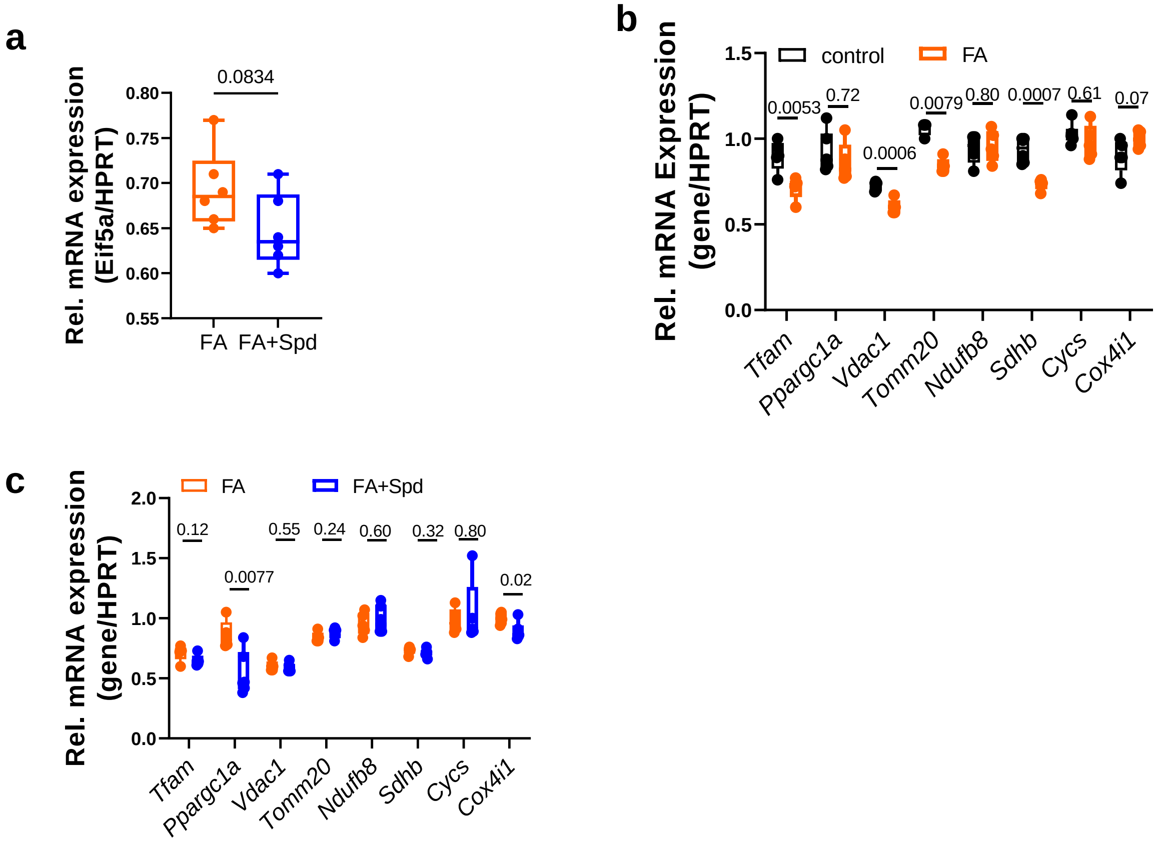

Fig. S7. FA decreased mRNA levels of some mitochondrial proteins, which were not rescued by spermidine co-treatment with FA in AML12 cells. (a) mRNA expression of Eif5a in FA- and FA+Spd-treated cells. (n=6). (b) mRNA expression of mitochondrial proteins in control and FA-treated cells. (n=5). (c) mRNA expression of mitochondrial proteins in FA- and FA+Spd-treated cells. (n=5). Cells were treated with FA (palmitic acid 0.6 mM, oleic acid 0.17 mM) with or without spermidine (100 µM) for 48 h. Data are shown as box-and-whisker with median (middle line), 25th–75th percentiles (box), and min–max values (whiskers). Significance was calculated by two-tailed Student's t-test or Mann-Whitney U -test, as appropriate. Source data are provided as a Source Data file.

Supplementary Figure 8

a

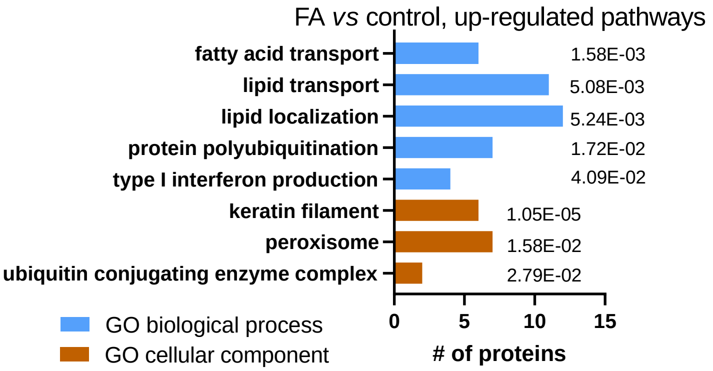

b

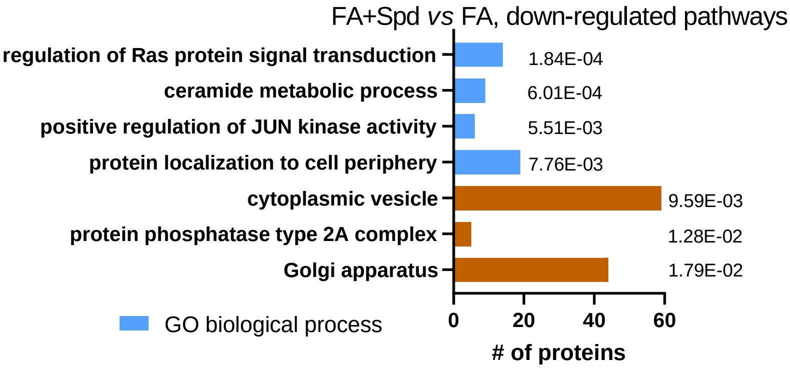

Fig. S8. Proteomics and gene ontology (GO) enrichment analysis (with corrected p-value indicated on the bar) of up-regulated proteins in FA *vs* control cells (a) and down-regulated proteins in FA+Spd vs FA cells (b). A Bonferroni correction was applied to correct for multiple testing. Source data are provided as a Supplementary Data file.

Supplementary Figure 9

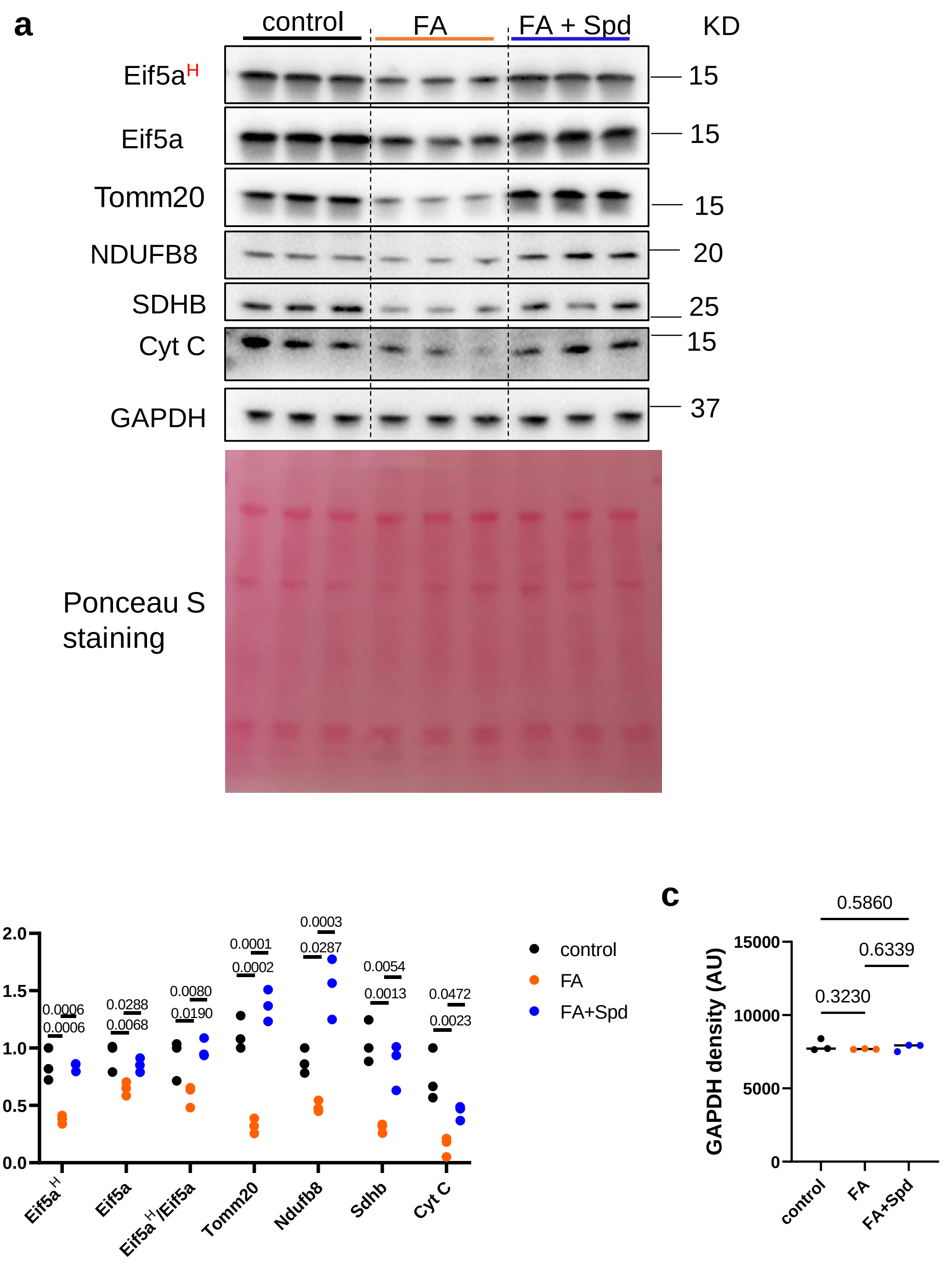

Fig. S9. FA decreased Eif5aH, and mitochondrial proteins, which can be rescued by co-treatment with spermidine in serum free medium. (a-b) Western blot (a) and densitometric analysis (b) of Eif5aH and mitochondrial proteins, (n=3), (c) GAPDH density in control, FA-, and FA+Spd-treated AML12 cells. (n=3). AML12 cells were plated in 6-well plate in FBS-containing medium. After attachment (overnight culture), the medium was replaced with FBS-free medium, and cells were treated with BSA-conjugated FA (palmitic acid 0.6 mM, oleic acid 0.17 mM) with or without spermidine (100 µM) for 48 h. Significance was calculated by One-Way ANOVA. Source data are provided as a Source Data file.

Supplementary Figure 10

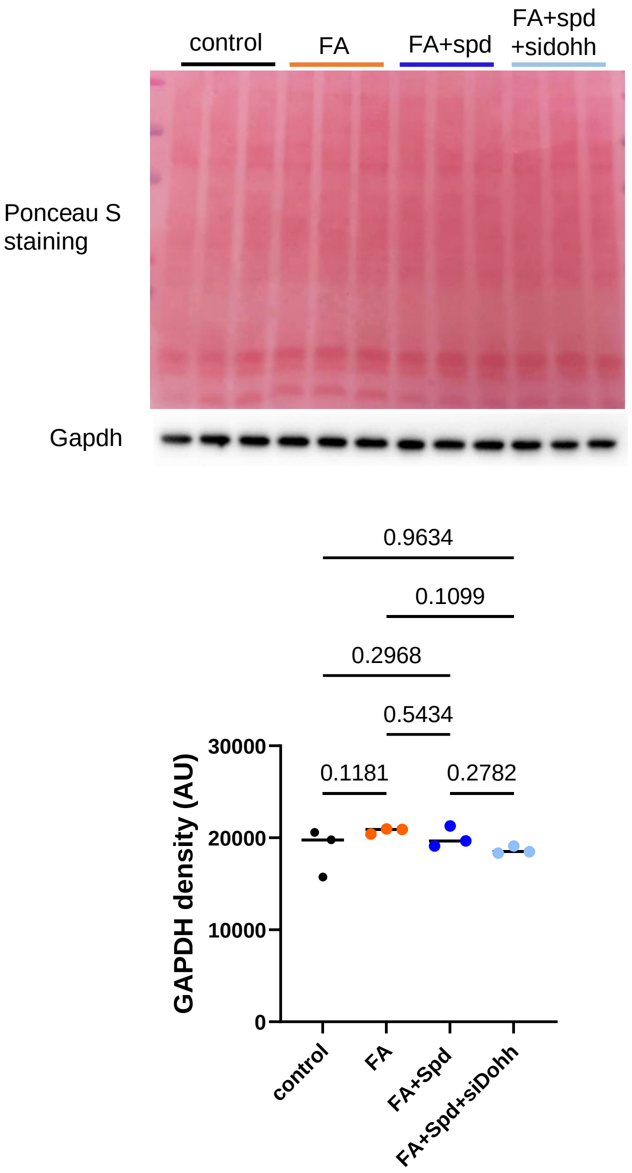

Fig. S10. Ponceau S staining of PVDF membrane after transfer, Western blot and density of GAPDH. AML12 cells were first transfected with 20 nM of negative (siNeg), or Dohh (siDohh) siRNA for 24 h, followed by treatment with BSA-conjugated FA (palmitic acid 0.6 mM, oleic acid 0.17 mM) with or without spermidine (100 µM) for 48 h. (n=3). Source data are provided as a Source Data file.

Supplementary Figure 11

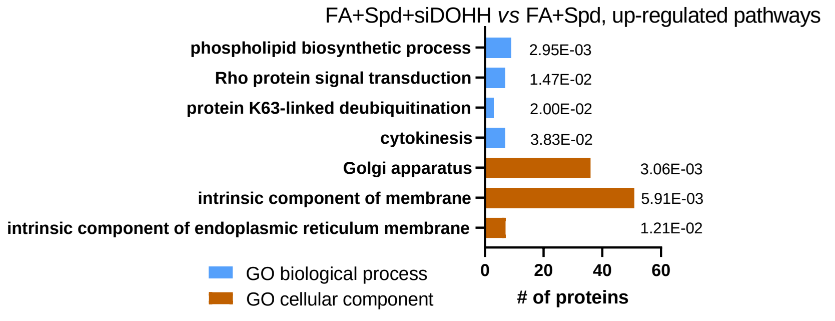

Fig. S11. Proteomics and gene ontology (GO) enrichment analysis (with corrected p-value indicated on the bar) of up-regulated proteins in FA+Spd+siDOHH *vs* FA+Spd cells. A Bonferroni correction was applied to correct for multiple testing. Source data are provided as a Supplementary Data file.

Supplementary Figure 12

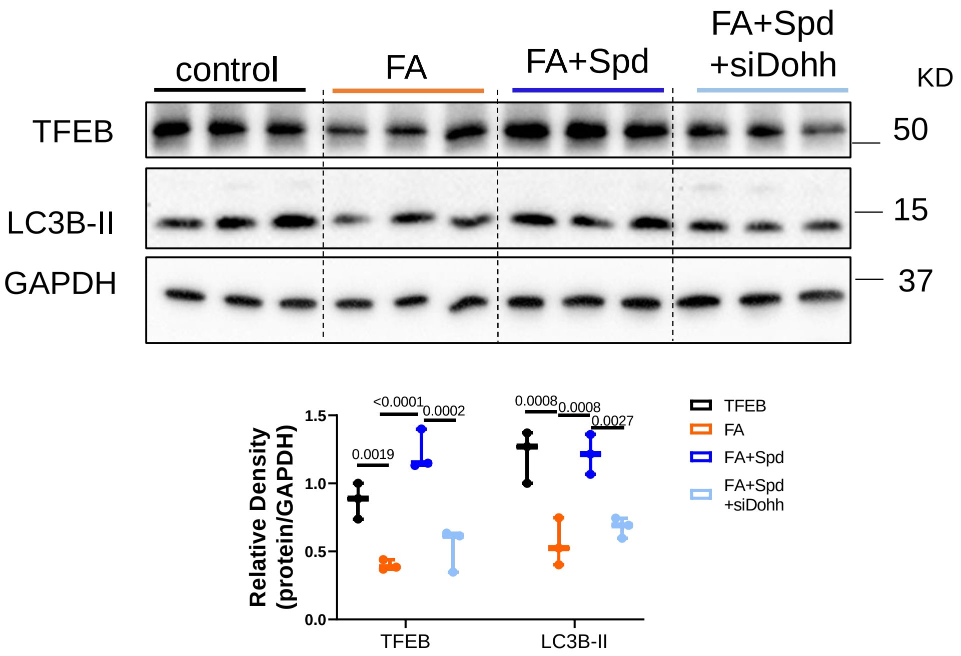

Fig. S12. Spermidine restored autophagy in FA-treated cell in Eif5a^{H}-dependent manner. Western blot and densitometric analysis of TFEB and LC3B-**II**. AML12 cells were first transfected with 20 nM of negative (siNeg), or Dohh (siDohh) siRNA for 24 h, followed by treatment with BSA-conjugated FA (palmitic acid 0.6 mM, oleic acid 0.17 mM) with or without spermidine (100 µM) for 48 h. (n=3). Data are shown as box-and-whisker with median (middle line), 25th–75th percentiles (box), and min–max values (whiskers). Significance was calculated by One-Way ANOVA. Source data are provided as a Source Data file.

Supplementary Figure 13

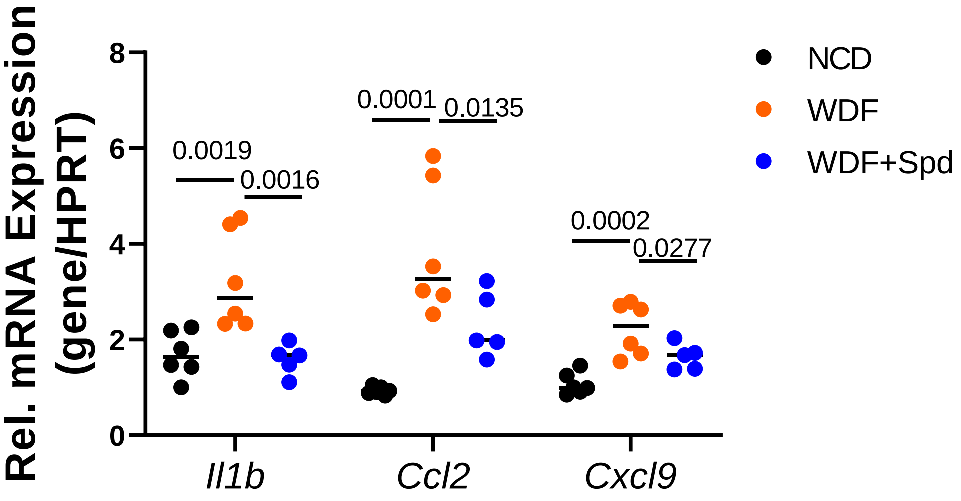

Fig. S13. Spermidine supplementation prevented hepatic inflammation. mRNA expression of inflammatory makers in the liver from mice fed on NCD (n=6), WDF (n=6), WDF+Spd (n=5) for 16 weeks. Significance was calculated by One-Way ANOVA. Source data are provided as a Source Data file.

Supplementary Figure 14

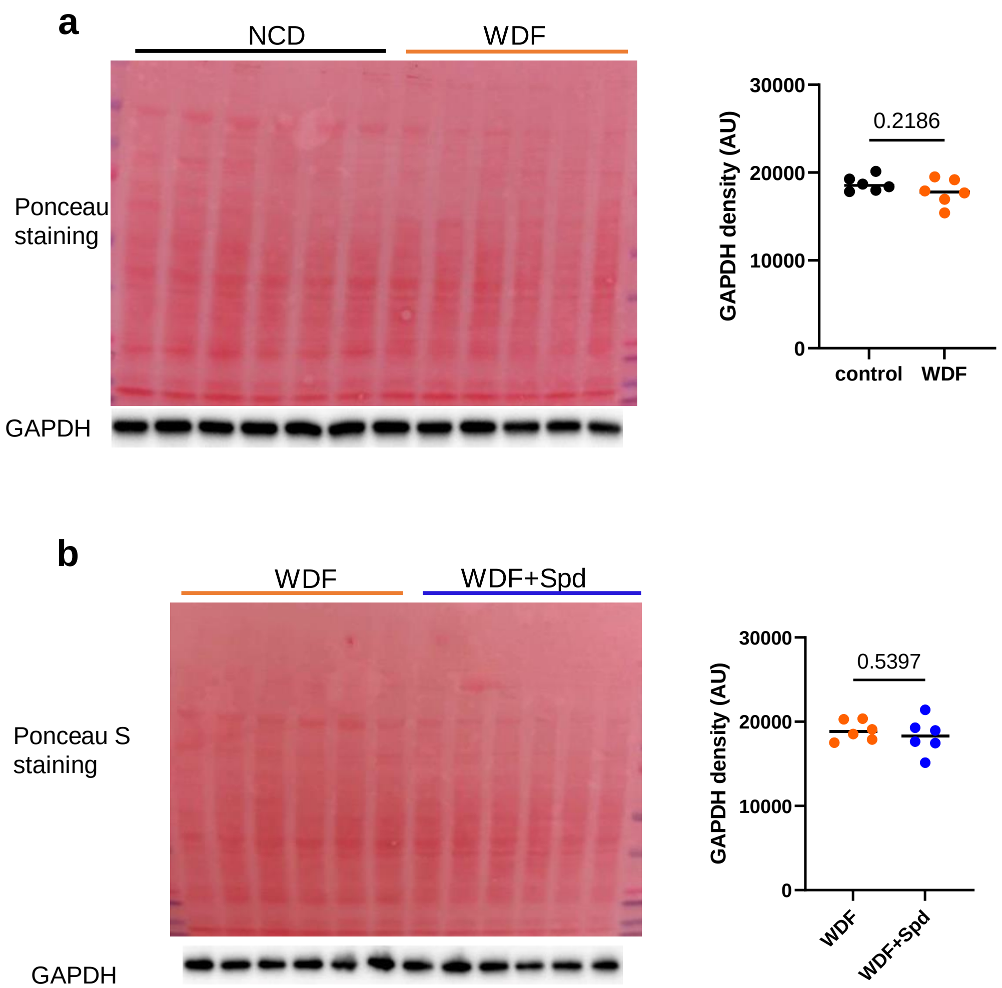

Fig. S14. Ponceau S staining of PVDF membrane after transfer, Western blot and density of GAPDH. (a) Liver protein lysate from mice fed on NCD (n=6) and WDF (n=6). (b) Liver protein lysate from mice fed on WDF (n=6) and WDF +Spd (n=6). Source data are provided as a Source Data file.

Supplementary Figure 15

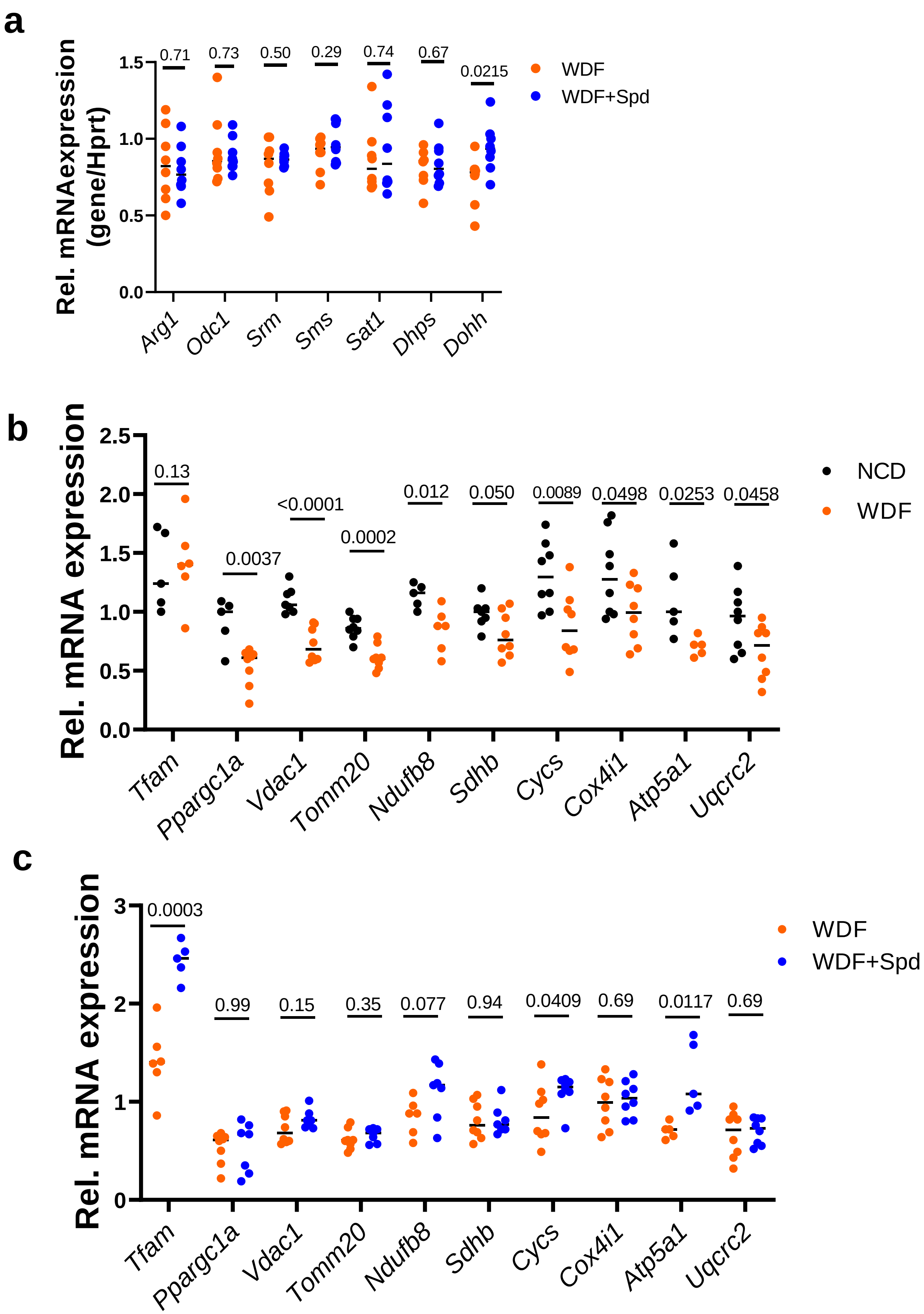

Fig. S15. Spermidine supplementation didn't show a major impact on WDF-decreased mRNA expression of polyamine biosynthesis enzymes and mitochondrial proteins. (a) Hepatic mRNA expression of mitochondrial proteins in NCD- *vs.* WDF-fed mice. (n=8). (b-c) Hepatic mRNA expression of polyamine biosynthesis enzymes (b) and mitochondrial proteins (c) in WDF- *vs.* WDF+Spd. (n=8). Significance was calculated by two-tailed Student's t-test or Mann-Whitney U-test, as appropriate. Source data are provided as a Source Data file.

Supplementary Table 1. Summary of differentially expressed proteins

Compared Samples	Number of proteins	regulated Type	fold-change>1.5
**siDohh vs control**	4286	up-regulated	297
		down-regulated	290
**FA vs control**	4272	up-regulated	254
		down-regulated	332
**FA+Spd vs FA**	4266	up-regulated	326
		down-regulated	361
**FA+Spd+siDohh vs FA+Spd**	4271	up-regulated	270
		down-regulated	301

The differentially expressed proteins were identified as fold change >1.5 fold and $p < 0.05$. P value was determined by significance A test.

Supplementary Table 2. Sequences of primers

mouse primers		
Dohh	Forward	CGATACCGTGCCATGTTTGC
	Reverse	GTCCTCAATGTGCTCTCGCA
Tfam	Forward	GAGTTCCCACGCTGGTAGTG
	Reverse	TGATAGACGAGGGGATGCGA
Ppargc1a	Forward	TCCTCTTCAAGATCCTGTTAC
	Reverse	CACATACAAGGGAGAATTGC
Vdac1	Forward	AGTGGGAAGAAGACCCCGAGA
	Reverse	AAGATCGGCGTATGTGGGAGG
Tomm20	Forward	CCTTGAAGAGATACAGCTTG
	Reverse	CAATTCTCTGACTAATGGTCG
Ndufb8	Forward	ACTCAGGATGAACTGGGGTG
	Reverse	AGCACGTAGAGGGAAGGACAA
Sdhb	Forward	CAGCTACTGGTGGAACGGAG
	Reverse	TGACACCAGAGTTGACAGGC
Cycs	Forward	CTACCACGGCTCTCCCTTTC
	Reverse	AGGTTTGAGGTGACATGCCC
Cox4i1	Forward	TACTTCGGTGTGCCTTCGAG
	Reverse	CCACATCAGGCAAGGGGTAG
Atp5a1	Forward	TGCCTTGACCTTCCTTTGCG
	Reverse	TTTTTGGAGACCAGTCCCGC
Uqcrc2	Forward	CCCATCTTGCTTTGCTGTCT
	Reverse	AGCCGATTCTTGACAGAGGAG
Il1b	Forward	GGATGATGATGATAACCTGC
	Reverse	CATGGAGAATATCACTTGTTGG
Ccl2	Forward	GAAGGAATGGGTCCAGACAT
	Reverse	ACGGGTCAACTTCACATTCA
Cxcl9	Forward	CGAGGCACGATCCACTACAA
	Reverse	AGGCAGGTTTGATCTCCGTT
Arg1	Forward	GGTCTGTGGGGAAAGCCAAT
	reverse	TGGTTGTCAGGGGAGTGTTG
Odc1	Forward	CAGAGCCATAGTGAGCACCC
	Reverse	ATGTGCTCTGGCGACTTTCA
Srm	Forward	TGGTGGACTACGCCTACTGT
	Reverse	GTGAACTCGGGCAGTACGAA
Sms	Forward	CGCCAAAGAAGGGAGAGAGTT
	Reverse	GGACAATACAGACGCCCCAG
Sat1	Forward	GCAGCAGTATGCACTTCTTGG
	Reverse	GGAATGGGTGCTCGCTCTAT
Dhps	Forward	CGGGAGACCATTCGATACCTC
	Reverse	CTGAACTCGCCAAGGTATGTG

Fig. S9

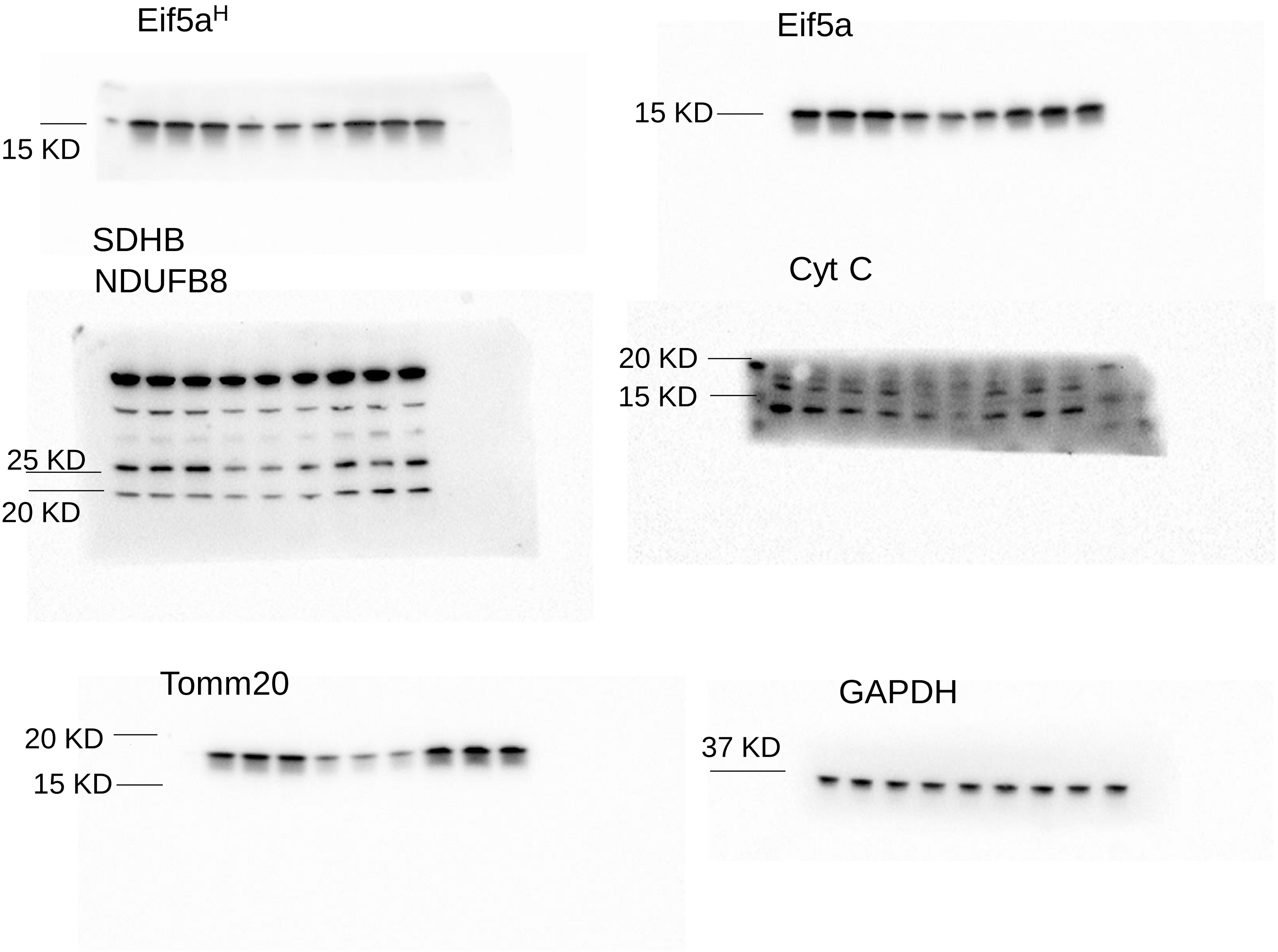

Eif5aH
15 KD
Eif5a
15 KD
SDHB
NDUFB8
25 KD
20 KD
Cyt C
20 KD
15 KD
Tomm20
20 KD
15 KD
GAPDH
37 KD

Fig. S12

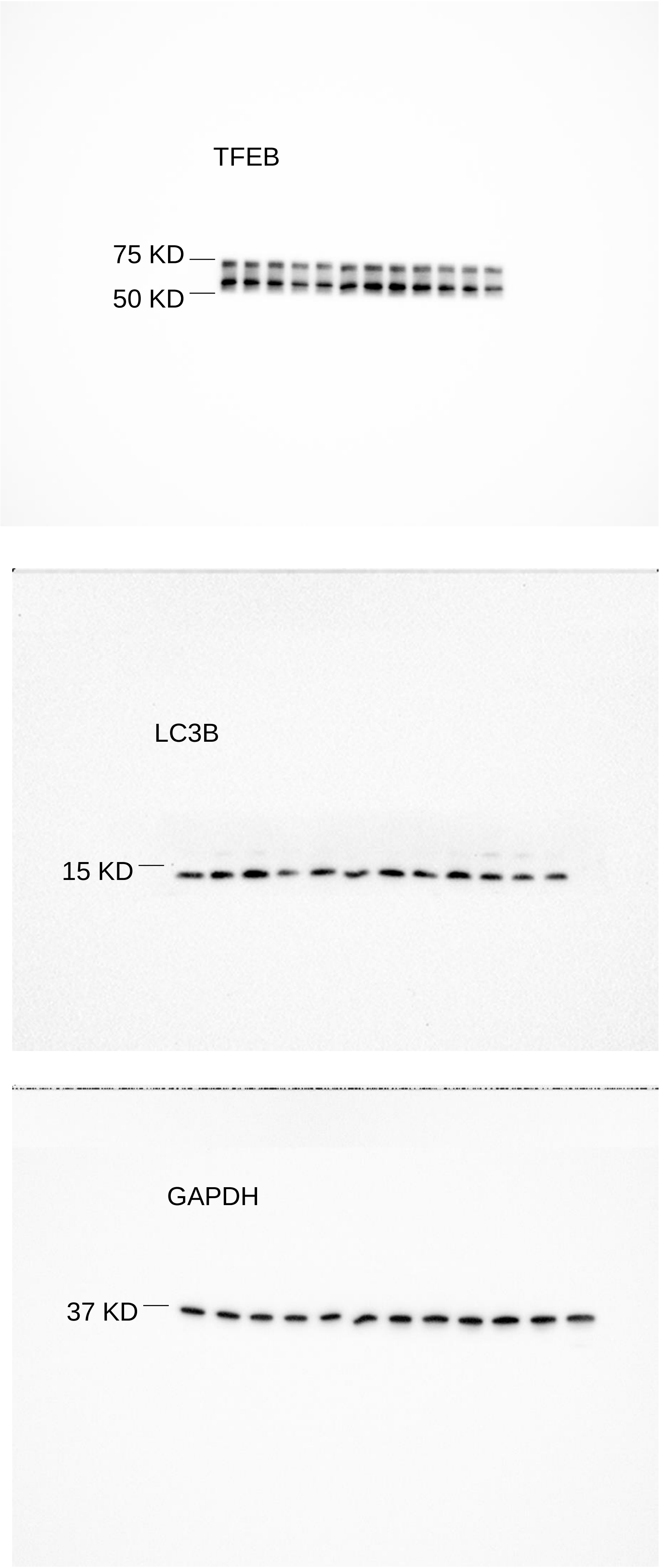

Supplement: Supplementary file 1 — Supplementary Information [file 41467_2022_32788_MOESM1_ESM.pdf]
