# Supplementary material for: Spermidine-mediated hypusination of translation factor EIF5A improves mitochondrial fatty acid oxidation and prevents non-alcoholic steatohepatitis progression
Source: Nat Commun. 2022 Sep 3;13:5202. doi: 10.1038/s41467-022-32788-x (PMC9440896; doi:10.1038/s41467-022-32788-x)
Supplement: Supplementary file 3 — Description of Additional Supplementary Files [file 41467_2022_32788_MOESM3_ESM.pdf]

Description of Additional Supplementary Files

File Name: Supplementary Data 1
Description: Gene ontology (GO) enrichment analysis of down-regulated proteins in Dohh KD (siDohh) vs control (siCtrl) AML12 cells. Pathway analysis of differentially expressed proteins were performed by WebGestalt. A Bonferroni correction was applied to correct for multiple testing.

File Name: Supplementary Data 2
Description: Gene ontology (GO) enrichment analysis of up-regulated proteins in Dohh KD (siDohh) vs control (siCtrl) AML12 cells. Pathway analysis of differentially expressed proteins were performed by WebGestalt. A Bonferroni correction was applied to correct for multiple testing.

File Name: Supplementary Data 3
Description: Gene ontology (GO) enrichment analysis of down-regulated proteins in fatty acids treated (FA) vs control (Ctrl) AML12 cells. Pathway analysis of differentially expressed proteins were performed by WebGestalt. A Bonferroni correction was applied to correct for multiple testing.

File Name: Supplementary Data 4
Description: Gene ontology (GO) enrichment analysis of up-regulated proteins in fatty acids treated (FA) vs control (Ctrl) AML12 cells. Pathway analysis of differentially expressed proteins were performed by WebGestalt. A Bonferroni correction was applied to correct for multiple testing.

File Name: Supplementary Data 5
Description: Gene ontology (GO) enrichment analysis of down-regulated proteins in fatty acids and spermidine treated (FA+Spd) vs fatty acids treated (FA) AML12 cells. Pathway analysis of differentially expressed proteins were performed by WebGestalt. A Bonferroni correction was applied to correct for multiple testing.

File Name: Supplementary Data 6
Description: Gene ontology (GO) enrichment analysis of up-regulated proteins in fatty acids and spermidine treated (FA+Spd) vs fatty acids treated (FA) AML12 cells. Pathway analysis of differentially expressed proteins were performed by WebGestalt. A Bonferroni correction was applied to correct for multiple testing.

File Name: Supplementary Data 7
Description: Gene ontology (GO) enrichment analysis of down-regulated proteins in fatty acids and spermidine treated AML12 cells with (FA+Spd+siDohh) or without (FA+Spd) Dohh KD. Pathway analysis of differentially expressed proteins were performed by WebGestalt. A Bonferroni correction was applied to correct for multiple testing.

File Name: Supplementary Data 8
Description: Gene ontology (GO) enrichment analysis of up-regulated proteins in fatty acids and spermidine treated AML12 cells with (FA+Spd+siDohh) or without (FA+Spd) Dohh KD. Pathway analysis of differentially expressed proteins were performed by WebGestalt. A Bonferroni correction was applied to correct for multiple testing.

File Name: Supplementary Data 9
Description: Lipidomic profiling employing liquid chromatography coupled with mass spectrometry (LC–MS) measurement of hepatic TAGs, diacylclycerols (DAGs), monoacylglycerols (MAGs), and cholesterol ester (CEs) levels in mice fed with NCD (n=6), WDF (n=8), and WDF+Spd (n=7).
